# Brainstem Astrocytes Regulate Estrus‐Dependent Oscillations in Food Intake

**DOI:** 10.1111/jnc.70498

**Published:** 2026-06-18

**Authors:** K. Selin Ozkaya, Ceyda Yalcin, Emily E. Haar, Ruchi Bhagat, Kirsteen N. Browning

**Affiliations:** ^1^ Department of Neuroscience and Experimental Therapeutics Penn State University ‐ College of Medicine Hershey Pennsylvania USA

## Abstract

Previous studies have shown that dorsal vagal complex (DVC) astrocytes play important roles in homeostatic regulation of food intake and caloric balance, specifically upregulation of NMDA receptor‐mediated glutamatergic signaling to brainstem dorsal motor nucleus of the vagus (DMV) motoneurons restores caloric balance following exposure to caloric dense diets. DMV neurons are critical to the regulation of gastric functions, including motility, tone, and emptying; hence food intake and energy homeostasis. Prior studies have also shown that caloric intake in female rats fluctuates across the estrus cycle, with food intake being lowest during periods of high estrogen levels. The aim of the current study was to investigate whether these estrus‐cycle dependent oscillations in food intake also involve DVC astrocyte adaptation. Immunohistochemical measurement of astrocytes across the estrus cycle uncovered an increase in glial‐fibrillary acidic protein immunoreactivity (GFAP‐IR) as well as an increase in astrocyte morphological complexity associated with high estrogen levels. Chemogenetic inhibition of DVC astrocytes eliminated food intake oscillations, whereas chemogenetic activation resulted in a consistent decrease in food intake, regardless of estrus status. Electrophysiological recordings from DMV neurons revealed that the estrus‐dependent decrease in food intake was associated with activation of DMV NMDA receptors, and pharmacological inhibition of either estrogen receptors or brainstem astrocytes prevented this mechanism. In contrast, application of estradiol uncovered astrocyte‐ and NMDA‐receptor dependent signaling to DMV neurons in low estrogen states. The findings of the present study demonstrate that DVC astrocytes and/or NMDA signaling play a fundamental role in estrogen‐dependent fluctuations in food intake and energy homeostasis.

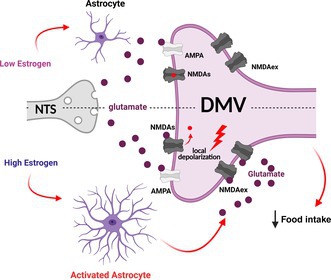

## Graphical abstract

DVC astrocytes regulate food intake and caloric balance via modulation of NMDA receptor‐mediated glutamatergic signaling to brainstem DMV motoneurons. In female rats, GFAP‐IR intensity and astrocyte morphological complexity increased during periods of high estrogen levels when food intake was decreased, and chemogenetic manipulation of DVC astrocytes eliminated these fluctuations in food intake. Electrophysiological recordings from DMV neurons revealed that the estrus‐dependent decrease in food intake was associated with activation of DMV NMDA receptors. The present study demonstrates that DVC astrocytes and/or NMDA signaling play fundamental roles in estrogen‐dependent fluctuations in food intake and energy homeostasis. Created with BioRender under license.

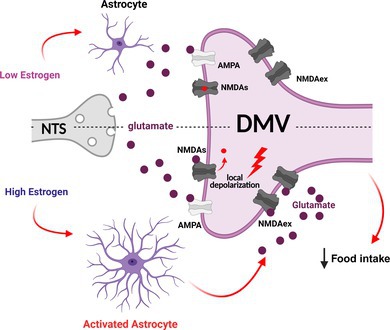

**Abbreviations**
DCZ: deschloroclozapine
DMV: dorsal motor nucleus of the vagus
DVC: dorsal vagal complex
FA: fluoroacetate
GFAP: glial fibrillary acidic protein
GI: gastrointestinal
HE: high estrogen
LE: low estrogen
mEPSC: miniature excitatory post synaptic currents
NMDA: N‐methyl‐D‐aspartate
NMDAR: N‐methyl‐D‐aspartate receptor
NMDARex: extrasynaptic N‐methyl‐D‐aspartate receptor
NTS: nucleus tractus solitarius
OVX: ovariectomy
RRID: Research Resource Identifier, see Scicrunch.Org
SEM: standard error of the mean

## 1 Introduction

Circulating ovarian hormones, particularly estrogen, play a critical role in shaping feeding behavior, as evidenced by well‐documented fluctuations in food intake across the estrus (Asarian and Geary 2006; Buffenstein et al. 1995; Lyons et al. 1989; Gong et al. 1989) and menstrual cycle (Blaustein and Wade 1976; Butcher et al. 1974; ter Haar 1972). Periods of higher estrogen levels are associated with a reduction in food intake and increased energy expenditure, while both experimental (e.g., ovariectomy) or physiological (e.g., menopause) reductions in estrogen induce hyperphagia and decrease energy expenditure (Blaustein and Wade 1976; Wallen et al. 2001; Martínez de Morentin Pablo et al. 2014; Mauvais‐Jarvis et al. 2013; López and Tena‐Sempere 2015). Although this relationship between estrogen and energy homeostasis is well established, the neural mechanisms through which estrogen dynamically modulates feeding remain to be elucidated.

While the hypothalamus has been the primary focus of investigations into the estrogen‐dependent suppression of food intake, brainstem neurocircuits also play critical roles in the regulation of homeostatic feeding behavior, particularly in the control of meal size and termination via vagally dependent neurocircuits that regulate gastrointestinal (GI) functions. Briefly, sensory information from the GI tract is relayed centrally through vagal afferent fibers to the nucleus tractus solitarius (NTS), which integrates these inputs along with signals from other brainstem and hypothalamic regions involved in autonomic control, before sending glutamatergic, GABAergic, and catecholaminergic projections to the dorsal motor nucleus of the vagus (DMV) neurons. DMV neurons are preganglionic parasympathetic pacemaking neurons that innervate the GI tract via the efferent vagus nerve to coordinate gastric functions, including tone, motility, and emptying, thereby regulating food intake and energy homeostasis. Previous studies have shown that brainstem astrocytes can enhance excitatory postsynaptic signaling to DMV neurons in response to an acute high‐fat diet challenge, ultimately restoring caloric homeostasis (Clyburn et al. 2021, 2023).

Brainstem astrocytes and neurons are well‐positioned to respond to circulating estrogen levels; not only do they express estrogen receptors, but the dorsal vagal complex (DVC; i.e., NTS, DMV, and area postrema) is a circumventricular organ whose fenestrated capillaries and leaky blood–brain barrier allow greater access of circulating factors, including gonadal hormones such as estrogen. This suggests that brainstem astrocytes, hence astroglial regulation of DMV neuronal excitability, may be differentially engaged by estrogen fluctuations across the estrus cycle although this has not been established. The aim of the present study, therefore, was to test the hypothesis that brainstem astrocytes play a fundamental and critical role in the regulation of estrogen‐dependent oscillations in food intake.

## 2 Methods

### 2.1 Ethics Statement

All experiments were approved by the Pennsylvania State University College of Medicine Institutional Animal Care and Use Committee (approval reference 47868) and conducted in accordance with the National Institutes of Health regulations. The reporting of animal experiments adheres to the principles and regulations for animal research ethics and complies with the ARRIVE guidelines.

### 2.2 Animals

Virgin female Sprague Dawley (SD) rats (*N* = 65, Charles River Laboratories) were used for electrophysiological (*N* = 29), immunohistochemistry (*N* = 26 for Sholl Analysis of which *N* = 20 were also used for mean fluorescence intensity analysis), and food intake studies (*N* = 10). Note that some rats were used in multiple experiments, for example, feeding studies and subsequent electrophysiology or immunohistochemistry. All rats had ad libitum access to water and a control chow diet (fat, protein, carbohydrate content: 14:27:59% kcal; Purina Mills). Rats were group housed in plastic ventilated cages on corn‐cob bedding housed in Penn State College of Medicine's humidity (30%–70%) and temperature (20°C–26°C) controlled Comparative Medicine facilities on an artificial 12:12 h light cycle. For food intake studies, rats were housed individually. Animals were 8–10 weeks old when in vitro experiments were conducted, and 10–12 weeks old when in vivo experiments were conducted.

### 2.3 Estrus Staging via Cytology

Estrus stage was determined via vaginal lavage cytology. After identifying the predominant cell type, diestrus and metestrus were classified as low estrogen (LE), while proestrus and estrus were classified as high estrogen (HE). Estrus staging was assessed daily throughout food intake studies and immediately before euthanasia for electrophysiology.

### 2.4 Immunohistochemical Studies

Rats in HE (*N* = 6 free cycling/*N* = 7 OVX + HE) or LE (*N* = 6 free cycling/*N* = 7 OVX + LE) stages were anesthetized deeply (loss of a foot‐pinch withdrawal reflex) with thiobutabarbital (Inactin; 150 mg/kg, i.p.) and perfused transcardially with 0.9% saline followed by 4% paraformaldehyde. The brains were removed and post‐fixed at 4°C for 3 days in 4% paraformaldehyde + 20% sucrose. The brainstems were mounted on a freezing microtome for sectioning at 50 μm throughout the rostro‐caudal extent of the DVC. Brainstem slices were stored at −20°C in long term storage buffer (0.1 M PBS, 30% sucrose, 30% ethylene glycol) until immunohistochemical processing.

Every fourth free‐floating brainstem slice was rinsed thoroughly in 0.1 M PBS and incubated in Immunobuffer (IB) (0.3% Triton X‐100 in Tris‐PBS) for 1 h, then IB + normal donkey serum (NDS; 1%; Abcam Cat# ab7475, RRID:AB_2885042, Cambridge, MA, USA) for 1 h. Slices were incubated in primary antibody for 3 days at room temperature on a shaker to identify astrocytes via the presence of glial fibrillary acid protein (GFAP; rabbit anti‐GFAP; 1:500 dilution, Millipore Cat# AB5804, RRID:AB_2109645) within the boundaries of the DMV as determined by the presence of choline acetyltransferase (ChAT; goat anti‐ChAT; 1:500 dilution, Millipore Cat# AB144P, RRID:AB_2079751). Following thorough washing in PBS, slices were incubated overnight in secondary antibodies (donkey anti‐rabbit Alexa Fluor 488; 1:500 dilution; Thermo Fisher Scientific Cat# A‐21206, RRID:AB_2535792 and donkey anti‐goat Alexa Fluor 568; 1:500 dilution; Thermo Fisher Scientific Cat# A‐11057, RRID:AB_2534104). The following day, after repeated washing in PBS, slices were mounted on subbed glass slides and cover‐slipped with Fluoromount G (Southern Biotechnology, Birmingham, AL, USA; RRID:SCR_015961).

### 2.5 Imaging and Analysis

Brainstem astrocytes were examined using a Zeiss LSM 900 compact microscope using Zen3.1 software. All images were taken at the same settings to allow for direct comparison between samples. Confocal images throughout the depth of the 50 μm‐thick slices were taken at 20× magnification and compiled into a z‐stack. The intensity of GFAP staining within the DMV was analyzed using ImageJ software (https://imagej.nih.gov/ij/; RRID:SCR_003070); background staining was normalized, and the mean pixel intensity was calculated.

Additional high magnification confocal images of GFAP‐immunoreactive (‐IR) astrocytes were taken and compiled to a z‐stack (one per DMV side per rat) to allow assessment of astrocyte morphology using Neurolucida imaging software (MBF Bioscience, Williston, VT; RRID:SCR_001775) and manually tracing in three dimensions (3‐D). Following the tracing, astrocytes were skeletonized and assessed with Sholl Analysis with concentric spheres at 5 μm intervals from the center of the cell body. The number of intersections at each distance was counted, and differences in process lengths, number of intersections, and number of branch points analyzed.

### 2.6 Chemogenetic DREADD Vector Transfection

Rats (*N* = 10) were anesthetized (ketamine: 80 mg/kg, Cat no. NDC 17033‐101‐10; acepromazine: 1 mg/kg, Cat no. NDC 0010‐3827‐01; xylazine: 10 mg/kg, Cat no. ANADA 200‐529; i.p.) until a deep level of anesthesia was obtained, and their core body temperature was kept at 37°C using a heating pad. They were placed on a stereotaxic frame (Kopf Instruments), and the brainstem was exposed via blunt dissection. Astrocytes were transfected by bilateral injections of adenoassociated virus expressing either the inhibitory DREADD (pAAV‐GFAP‐hM4D(Gi)‐mCherry, “GFAP‐hM4”; RRID:Addgene_50479) or excitatory DREADD (pAAV‐GFAP‐hM3D(Gq)‐mCherry, “GFAP‐hM3”; RRID:Addgene_50478). A borosilicate micropipette was used to microinject GFAP‐hM4 or GFAP‐hM3 (120 nL per injection site; viral titers 1 × 10^13^, diluted 1:10 in PBS at the time of use) at the following co‐ordinates (in mm from calamus scriptorius): −0.2 to +0.4 rostro‐caudal; ± 0.2–0.4 medio‐lateral from midline; and −0.6‐0.65 dorso‐ventral from the surface of the brainstem. Following recovery from anesthesia, rats were returned to their home cage for 3 weeks to allow for recovery and virus expression before performing any experiments involving chemogenetic manipulations. Rats were given carprofen (Zoetis, Troy Hills, NJ, USA; 1 mg/kg, s.c.) for pain management peri‐ and post‐operatively, every 24 h for 3 days. Rats were monitored daily for 5 days post‐surgery, with body weight and body condition score noted, as well as visual inspection of incision site.

### 2.7 Food Intake Measurements

A week after chemogenetic transfection surgery, rats (*N* = 10, GFAP‐hM4 = 5, GFAP‐hM3 = 5) were single‐housed. After 3 days acclimation to single housing, food intake and body weight were measured daily within 2 h of lights‐on. The estrus stage was assessed in the morning. Rats received saline injections (0.9%; 1 mL/kg intraperitoneally [i.p.]) in the morning and evening within 2 h of lights on/off for 7 days to allow for handling and injection acclimation. After measuring food intake across 3 cycles to establish a baseline, all rats received twice‐daily administration of the DREADD agonist deschlorocloazapine (DCZ; 0.1 mg/kg, i.p.; HelloBio, Cat no. HB8555). At the end of the study, rats were either anesthetized with thiobutabarbital (Inactin; 150 mg/kg, i.p.) and euthanized via transcardial perfusion‐fixation with 0.9% saline, followed by 4% paraformaldehyde (*N* = 4), while the remainder (*N* = 6) were used for electrophysiological recordings.

### 2.8 Ovariectomy and Estrogen Replacement Therapy

Rats (*N* = 14) were anesthetized with 2.5% isoflurane delivered in a 95% O_2_/5% CO_2_ gas mixture using a Vapomatic system. Following a midline incision to expose the abdominal cavity, the oviducts were ligated at both ends with braided silk suture (SP115, Lot: AAER994) and then transected to minimize bleeding. This procedure was performed bilaterally to achieve a complete ovariectomy. Core body temperature was maintained at 37°C throughout surgery and recovery by placing the animals on a heating pad.

During the same procedure, estrogen levels were replaced via implantation of a capsule containing 17β‐estradiol dissolved in sesame oil at either a high (*N* = 7; 180 μg/mL) or low (*N* = 7; 18–36 μg/mL) level. The vehicle capsule was obtained by only filling the silica tube with sesame oil (Strom et al. 2012). Pre‐made capsules were implanted subcutaneously through an incision made at the dorsal neck region. The incision was sutured, and animals were allowed to recover for 2 weeks prior to experimentation. Rats were given carprofen (Zoetis, Troy Hills, NJ, USA; 1 mg/kg, s.c.) for pain management peri‐ and post‐operatively, every 24 h for 3 days. Rats were monitored daily for 5 days post‐surgery, with body weight and body condition score noted, as well as visual inspection of incision site.

### 2.9 Serum Estradiol Measurement by ELISA

Rats were anesthetized with isoflurane (5% in air) before blood collection via cardiac puncture at the time of euthanasia; blood was allowed to clot at room temperature for 2 h before centrifugation at 4°C (Eppendorf Centrifuge 5417R; RRID:SCR_019847; 2000*g* for 5 min) to obtain serum. Samples were stored at −20°C until use. Serum/plasma estradiol concentrations were measured using the rat estradiol ELISA kit (Thermofisher Scientific, Cat no. EELR015) according to the manufacturer's instructions. Samples from HE (*N* = 11), OVX + HE (*N* = 4), LE (*N* = 10), and OVX + LE (*N* = 4) rats were assayed in triplicate at 1:4 dilution (112.6 pg/mL sensitivity). The optical density of the samples was measured at 450 nm using the Bio‐Rad iMark Microplate Reader (RRID:SCR_023799). A standard curve was generated using a four‐parameter logistic (4PL) model with an online immunoassay analysis tool (www.myassays.com; RRID:SCR_016562), and sample estradiol concentrations were determined by interpolation from this curve. Values were corrected for any dilution factor and reported as pg/mL.

### 2.10 Brainstem Removal for Electrophysiological Recordings

Rats were anesthetized with isoflurane (5% in air) before being euthanized via administration of a bilateral pneumothorax. The brainstem was removed and sliced as previously described (Clyburn et al. 2023; Carson et al. 2023). Briefly, the brainstem was removed and submerged in ice‐cold, oxygenated high sucrose Krebs' solution (in mM: 87.0 NaCl, 2.5 KCl, 0.5 CaCl_2_, 7.0 MgCl_2_, 1.25 NaH_2_PO_4_, 25.0 NaHCO_3_, 25.0 d‐glucose, and 75.0 sucrose). The brainstem was then mounted on a vibratome, and coronal sections were cut at 300 μm throughout the rostro‐caudal extent of the DVC. Slices were placed in oxygenated, warm (30°C), high sucrose Krebs' solution for 30 min, then transferred to a 50:50 mixture of warm normal Krebs' solution (in mM NaCl, 25 NaHCO_3_, 2.5 KCl, 1.2 MgCl_2_, 2.4 CaCl_2_, 1.2 NaH_2_PO_4_, and 10 d‐glucose, maintained at pH 7.4 by bubbling with 95% O_2_/5% CO_2_) and high sucrose Krebs' solution. After that, slices were kept in normal Krebs' solution for another 30 min before recording.

### 2.11 Electrophysiological Recordings

Brainstem slices were placed in a perfusion chamber mounted on the stage of a Nikon E600FN microscope, which was equipped with a tetramethylrhodamine isothiocyanate (TRITC) epifluorescent filter. Slices were perfused with warm Krebs' solutions at a rate of 2.0–2.5 mL/min. When recording from GFAP‐hM3 or GFAP‐hM4 transfected rats, viral injection sites were confirmed based on the presence of fluorescent mCherry‐labeled astrocytes, and electrophysiological recordings were made from medial DMV neurons surrounded by mCherry‐labeled astrocytes or from medial DMV neurons in naïve brainstem slices. Patch pipettes (2–4 MΩ) were filled with a potassium gluconate intracellular solution (in mM: 128 potassium gluconate, 10 KCl, 0.3 CaCl_2_, 1 MgCl_2_, 10 HEPES, 1 EGTA, 1 NaATP, and 0.25 NaGTP adjusted to pH 7.35). A single‐electrode voltage clamp amplifier (Axopatch 200B, RRID:SCR_018866) was used, and data filtered at 2 kHz, digitized through a Digidata 1440 Interface, and stored and analyzed on a PC using pClamp 10 software (Molecular Devices, RRID:SCR_011323).

Glutamatergic miniature excitatory postsynaptic currents (mEPSCs) were measured in DMV neurons voltage clamped at −60 mV in the presence of tetrodotoxin (TTX; 1 μM) and bicuculline (BIC; 30 μM). The effects of perfusion with the NMDAR‐selective antagonist (2R)‐amino‐5‐phosphopentanoic acid (AP5; 25 μM) on the amplitude, frequency, and charge transfer of mEPSCs in LE and HE DMV neurons were assessed using MiniAnalysis software (Synaptosoft, Leonia, NJ, USA; RRID:SCR_002184). The effects of AP5 were assessed following superfusion with the astroglial metabolic inhibitor, fluoroacetate (FA; 100 μM), the extrasynaptic NMDAR‐selective antagonist, memantine (Mem; 30 μM), β‐estradiol (100 nM), the ERα antagonist, ICI182,780 (10 μM), and the DREADD agonist, descholoroclozapine (DCZ; 0.1 μM; 10 min). All drugs were applied until the response reached a plateau or at least for 5 min if no response was observed. TTX was purchased from Cayman Chemical Company, DCZ was purchased from HelloBio, and all other drugs were purchased from Millipore Sigma.

### 2.12 Statistical Analysis

No randomization was performed to allocate subjects in the current study. Study sample size was based upon previous studies of a similar nature (Clyburn et al. 2021, 2023). All statistical analysis was conducted using Prism version 10.6.1 (GraphPad, Boston, MA, USA; RRID:SCR_002798). For electrophysiological results, the response of any neuron was assessed before and after drug application with a paired, two‐tailed Student's *t*‐test. For comparisons across groups for an individual drug, one‐way ANOVA with post hoc Bonferroni test was used, while the intergroup comparisons were made using the Chi‐squared test. Responsivity of a neuron was determined by a change of more than 25% in its mEPSC properties (frequency, charge transfer, or amplitude). All results, both responsive and nonresponsive, are reported as the mean ± SEM, with exact *p*‐values. The significance is defined as *p* ≤ 0.05. While it is common practice within the field to record from neurons taken from multiple different brainstem sections in experimental animals, we recognize that these observations are not fully independent and the nested structure of the data is an important consideration when interpreting the results.

For caloric intake studies, the data were normalized to body weight, expressed as a percentage of baseline (average intake over the first 4 days) or as a percentage relative to LE levels. HE food intake was normalized to LE within each animal (LE = 100%) to enable within‐animal comparisons and account for inter‐individual variability. This normalization did not affect data distribution. Data are presented as daily (24‐h) caloric intake. The two‐tailed paired Student's *t*‐test was used to compare food intake across the estrus cycle in rats with chemogenetic brainstem astrocyte transfections before and after DCZ administration. The data are expressed as mean ± SEM, with the exact *p* value indicated, with significance denoted as *p* ≤ 0.05.

For chemogenetic transfection experiments, pre‐determined exclusion criteria included off‐target injection sites. One animal was excluded (GFAP‐hM4) post hoc due to an off‐target injection site, and one animal died (GFAP‐hM3) in the immediate post‐surgical period. No animals were replaced.

For astrocyte morphological analyses, individual reconstructed astrocytes were treated as units of analysis (*N* = 43 for LE, *N* = 46 for HE). Sholl parameters, including process length, number of intersections and branch points, were quantified for each astrocyte using the NeuroLucida software. To assess the potential influence of the nested nature of data resulting from multiple astrocytes from individual animals, additional inferential analyses were performed using animal level averages. Specifically, measurements from all reconstructed astrocytes within each animal were averaged to generate a single value per animal (*N* = 13 rats/group). Statistical comparisons based on these animal level averages remained significant for number of process intersections (AUC: 102.4 ± 12.89 LE vs. 268.1 ± 28.30 HE, two‐tailed unpaired Welch's *t*‐test *t*(16.77) = 5.329, *p* = < 0.0001), number of branch points (AUC: 28.50 ± 6.97 LE vs. 53.71 ± 6.83 HE, two‐tailed unpaired Welch's *t*‐test *t*(23.99) = 2.582, *p* = 0.0164) and process lengths (AUC: 686.5 ± 93.75 μm LE vs. 1793 ± 175.9 μm HE, two‐tailed unpaired Welch's *t*‐test *t*(18.31) = 5.553, *p* = < 0.0001) with those obtained from the astrocytes level analyses.

The Shapiro–Wilk test and Q–Q plots were used to assess normality assumptions. Minor deviations from normality were observed in the immunohistochemistry data; however, these were considered acceptable and Welch's *t*‐test was applied. Results were confirmed using the Mann–Whitney *U* test, which yielded comparable outcomes. Outliers were identified using the ROUT method (*Q* = 1%) and excluded from the analysis (LE intersections: *n* = 1; LE branch points *n* = 6; HE branch points *n* = 6; LE length: *n* = 2). All other datasets met normality assumptions.

## 3 Results

### 3.1 Experimental Timeline

**FIGURE 1:**
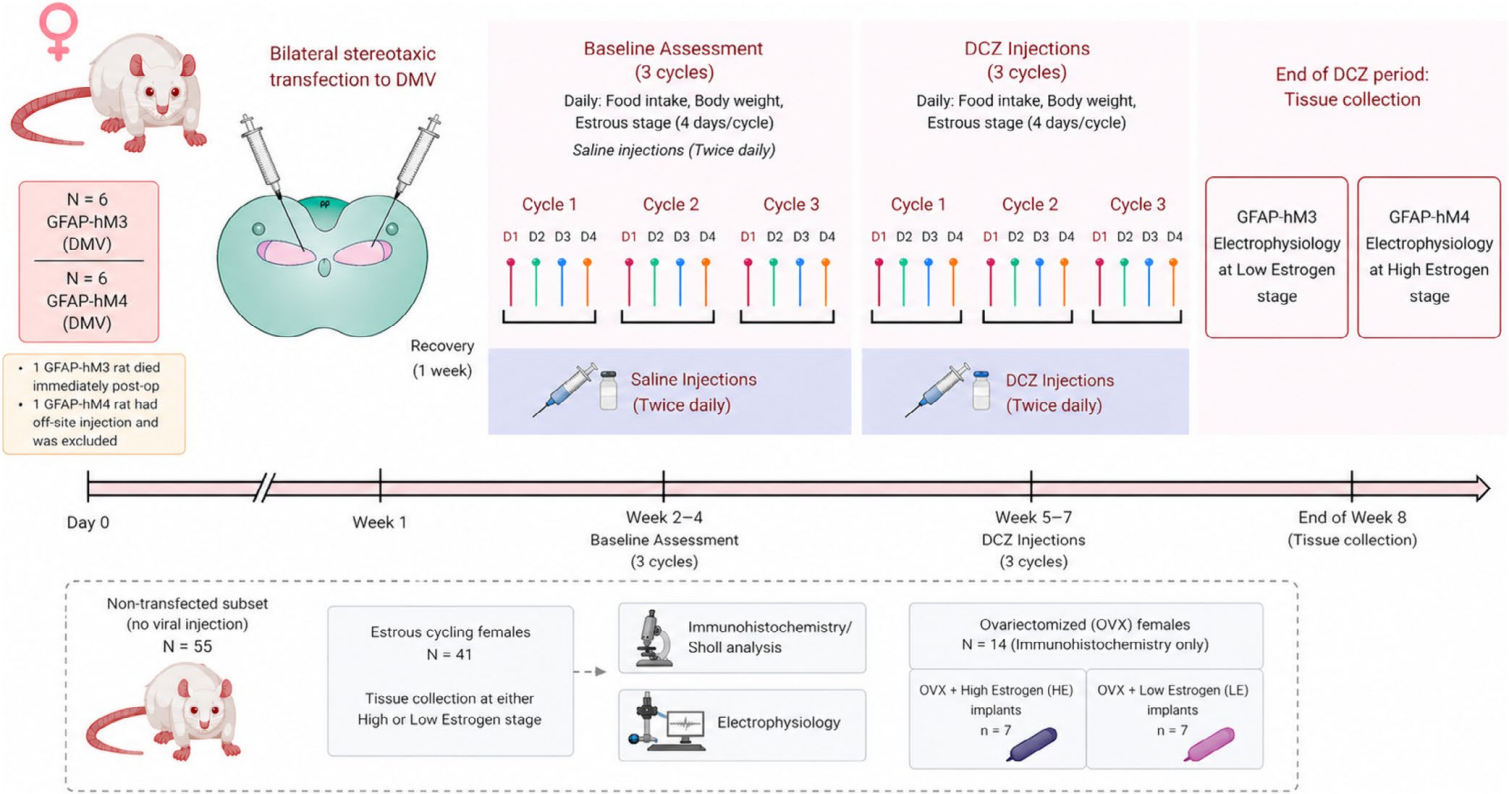
Experimental timeline for DMV astrocyte manipulation, electrophysiology, and Sholl analysis across estrous stages. Female rats received bilateral viral transfection of DREADDs targeting the DMV with either GFAP‐hM3 (*N* = 6) or GFAP‐hM4 (*N* = 6). Following a 1 week recovery, animals underwent baseline food intake assessment across three estrus cycles (4 days/cycle), during which estrus stage, food intake, and body weight were monitored daily. During the final 7 days of baseline assessment, animals received saline injections twice daily to habituate to handling and injections. Animals then received DCZ injections twice daily for an additional 3 estrus cycles, with continued daily monitoring. At the end of the paradigm, animals were used for electrophysiology: GFAP‐hM3‐expressing rats were assessed during the low estrogen stage, whereas GFAP‐hM4‐expressing rats were assessed during the high estrogen stage. A separate cohort of non‐transfected animals (*N* = 55) was used for electrophysiology and immunohistochemistry/Sholl analysis. Free‐cycling females (*N* = 41) were sacrificed during either high or low estrogen stages for immunohistochemistry/Sholl analysis or electrophysiology. OVX animals (*N* = 14) received either high (*N* = 7) or low (*N* = 7) estrogen implants and were used for immunohistochemistry/Sholl analysis. Created with Sci‐Draw under license.

The experimental timeline, including the temporal pattern of feeding studies, chemogenetic manipulation, as well as electrophysiological and immunohistochemical assessments is outlined in Figure 1.

### 3.2 Estrogen Level Measurement

Circulating serum estrogen levels did not differ between free cycling and ovariectomized animals under equivalent hormone conditions (HE 52.61 ± 1.97 pg/mL vs. OVX + HE 63.14 ± 5.39 pg/mL, one‐way ANOVA, *F*(3, 25) = 20.58, *p* = < 0.0001, post hoc Bonferroni's multiple comparisons test, *p* = 0.0824; LE 35.85 ± 1.78 pg/mL vs. OVX + LE 39.39 ± 2.81 pg/mL, one‐way ANOVA, *F*(3, 25) = 20.58, *p* = < 0.0001, post hoc Bonferroni's multiple comparisons test, *p* = > 0.9999), indicating that the estrogen replacement paradigm effectively reproduced the intended hormonal state and that group assignments via vaginal lavage were accurate. In contrast, estrogen levels were significantly different in high and low estrogen groups (HE 52.61 ± 1.97 pg/mL vs. LE 35.85 ± 1.78 pg/mL, one‐way ANOVA, *F*(3, 25) = 20.58, *p* = < 0.0001, post hoc Bonferroni's multiple comparisons test, *p* = < 0.0001; HE 52.61 ± 1.97 pg/mL vs. OVX + LE 39.39 ± 2.81 pg/mL, one‐way ANOVA, *F*(3, 25) = 20.58, *p* = < 0.0001, post hoc Bonferroni's multiple comparisons test, *p* = 0.0161; OVX + HE 63.14 ± 5.39 pg/mL vs. LE 35.85 ± 1.78 pg/mL, one‐way ANOVA, *F*(3, 25) = 20.58, *p* = < 0.0001, post hoc Bonferroni's multiple comparisons test, *p* = < 0.001; OVX + HE 63.14 ± 5.39 pg/mL vs. OVX + LE 39.39 ± 2.81 pg/mL, one‐way ANOVA, *F*(3, 25) = 20.58, *p* = < 0.0001, post hoc Bonferroni's multiple comparisons test, *p* = 0.0003; Figure S1). Together, these results confirm that estrogen levels in freely cycling rats classified as HE or LE based on cytology assessment via vaginal lavage are comparable to those observed in OVX animals receiving corresponding hormone replacement.

### 3.3 Caloric Intake Oscillates Across the Estrus Cycle

**FIGURE 2:**
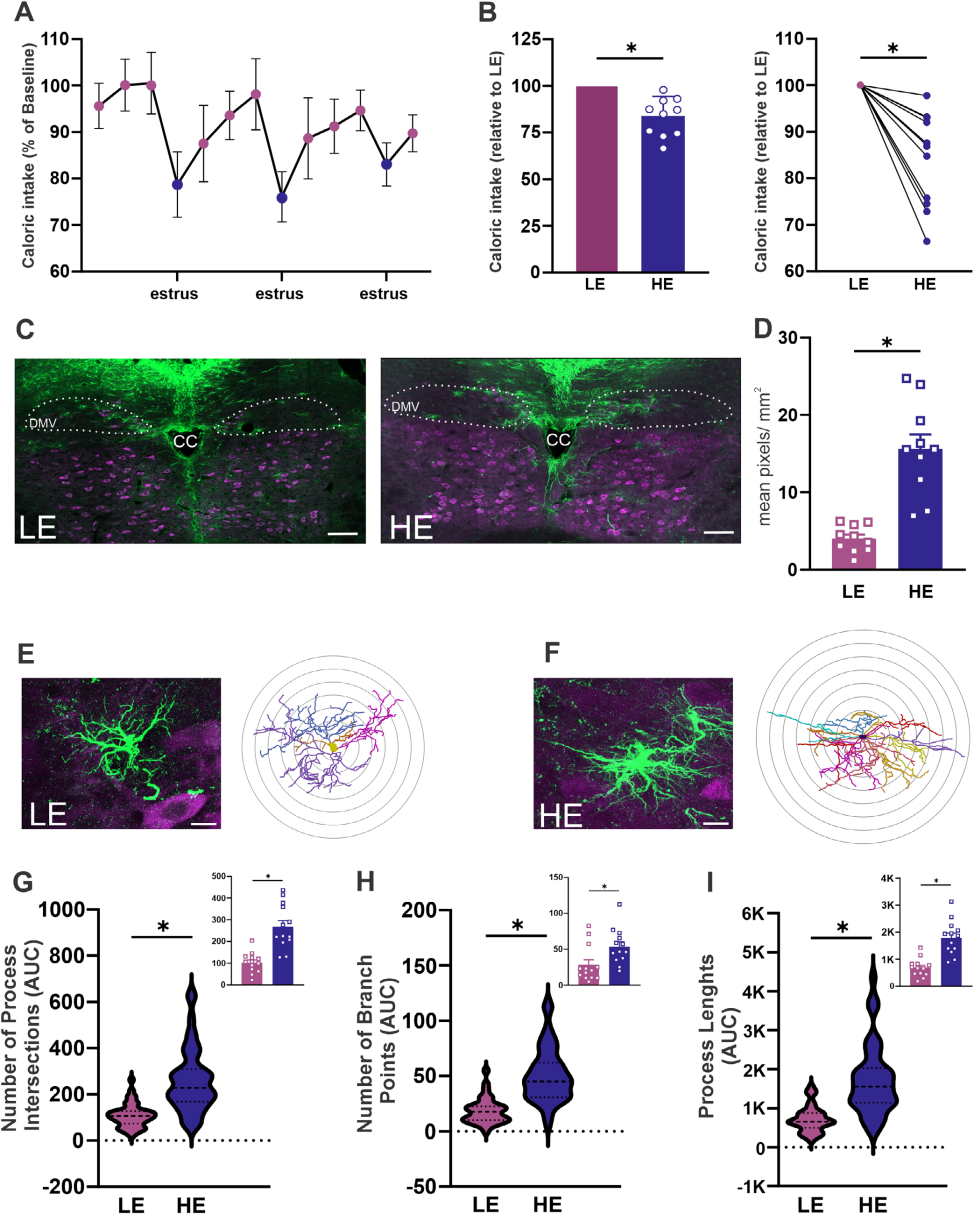
Food intake is reduced during elevated levels of estrogen and is associated with alterations in brainstem astrocytes. (A) Graphical summary of mean daily food intake in freely cycling rats across three consecutive 4‐day estrus cycles showing decreased intake during estrus. Rats were weighed, food intake was calculated, and the estrus staging was recorded daily throughout the study (*N* = 10 rats). (B) Graphical summary of caloric intake during LE and HE stages of the estrus cycle per animal (*N* = 10 rats), expressed as percentage relative to LE intake. (C) Representative images of LE and HE brainstem slices containing DMV (delineated with ChAT [outlined; magenta]) and stained with GFAP to identify astrocytes (green), illustrating astrocyte adaptation during HE. Scale bar = 100 um. (D) Quantification of GFAP fluorescence intensity (*N* = 10 rats/group) showing a significant increase in DMV astrocytes in HE rats. (E, F) Representative skeletonized DMV astrocytes and associated Sholl analysis of LE astrocyte (E) and HE astrocyte (F). Scale bar = 10 μm. (G) Area under the curve (AUC) of process intersections, (H) number of branch points and (I) process lengths (μm) within 60 μm of the soma, determined by Sholl analysis in LE (*N* = 13 rats/43 astrocytes) versus HE astrocytes (*N* = 13 rats/46 astrocytes). Violin plots display individual astrocytes, while inset histograms (upper right) show the mean AUC values pooled by subject. Note that HE astrocytes exhibited significantly greater process length, number of intersections and number of branch points compared to the LE, indicating increased morphological complexity consistent with activated astrocytes. All data are represented as mean ± SEM. (*p < 0.05, two‐tailed unpaired Welch's *t*‐test).

Food intake and body weight were measured in 10 virgin female rats, and the estrus stage was assessed daily via vaginal lavage. As previously (Drewett 1973, 1974; Eckel et al. 2000; Eckel 2004; Tarttelin and Gorski 1971), caloric intake was reduced significantly in all rats when estrogen levels increased (84.1% ± 3.6% relative to LE, HE caloric intake vs. LE caloric intake two‐tailed paired Student's *t*‐test, *t*(9) = 5.203, *p* = 0.0006, Figure 2A,B).

### 3.4 Brainstem Astrocytes Morphology Is Altered Across the Estrus Cycle

Immunofluorescent images of LE/OVX + LE and HE/OVX + HE rats (*N* = 20 animals; *N* = 6 HE; *N* = 4 OVX + HE; *N* = 6 LE; *N* = 4 OVX + LE) were used to assess GFAP‐IR within the DMV (Figure 2C). Since there was no difference in mean intensity of GFAP‐IR in HE versus OVX + HE (HE 14.96 ± 2.40 mean pixels/mm^2^ vs. OVX + HE 16.58 ± 3.45 mean pixels/mm^2^, two‐tailed unpaired Welch's *t*‐test, *t*(5.79) = 0.3851, *p* = 0.7139) and in LE vs. OVX + LE (LE 4.602 ± 0.7171 mean pixels/mm^2^ vs. OVX + LE 3.099 ± 0.7043 mean pixels/mm^2^, two‐tailed unpaired Welch's *t*‐test, *t*(7.566) = 1.495, *p* = 0.1753), the groups were pooled for subsequent analyses and hereafter designated as HE and LE. There was also no differences in mean intensity of GFAP‐IR in left versus right DMV (HE left 16.89 ± 1.72 mean pixels/mm^2^ vs. HE right 15.10 ± 2.10 mean pixels/mm^2^, two‐tailed paired Student's *t*‐test *t*(9) = 1.626, *p* = 0.1384; LE left 4.058 ± 0.61 mean pixels/mm^2^ vs. LE right 3.943 ± 0.50 mean pixels/mm^2^, two‐tailed paired Student's *t*‐test *t*(9) = 0.4494, *p* = 0.6638), the results were combined.

GFAP‐IR mean fluorescence intensity was significantly higher during HE compared to LE states (15.61 ± 1.89 HE vs. 4.00 ± 0.55 LE mean pixels/mm^2^, two‐tailed unpaired Welch's *t*‐test *t*(10.49) = 5.989, *p* = 0.0001; Figure 2D).

To confirm changes in astrocyte morphology (*N* = 13 rats per group [6 cycling, 7 OVX]; HE: 46 astrocytes [11 caudal, 25 intermediate, 10 rostral]; LE: 43 astrocytes [11 caudal, 22 intermediate, 10 rostral]) were analyzed via Sholl analysis of high magnification z‐stacked confocal images using NeuroLucida software to determine the process length, number of intersections, and the branch points of each astrocyte (Figure 2E,F). Differences were not observed in any morphological parameter in rostral versus intermediate versus caudal astrocytes and results were, therefore, combined. The number of process intersections (AUC: 108.1 ± 7.75 LE vs. 258.5 ± 19.91 HE, two‐tailed unpaired Welch's *t*‐test *t*(58.20) = 7.042, *p* = < 0.0001; Figure 2G), number of branch points (AUC: 18.43 ± 1.94 LE vs. 51.00 ± 3.81 HE, two‐tailed unpaired Welch's *t*‐test *t*(57.44) = 7.614, *p* = < 0.0001; Figure 2H) and the process lengths (AUC: 719.1 ± 51.82 μm LE vs. 1721 ± 131.3 μm HE, two‐tailed unpaired Welch's *t*‐test *t*(58.51) = 7.100, *p* = < 0.0001; Figure 2I) per astrocyte was significantly higher in HE compared to LE. Overall, within 60 μm from the center of the cell body, when in HE state, astrocytes had an increased number of processes, branch points, and process lengths. These results suggest that DMV astrocytes vary across the estrus cycle in an estrogen‐dependent manner.

To further confirm estrogen‐dependent alterations in astrocyte morphology, Sholl analysis was also completed on 44 astrocytes from 14 rats that underwent ovariectomy (OVX) and supplementation with high estrogen (*N* = 7 rats/23 astrocytes), or low estrogen (*N* = 7/21 astrocytes). There were no differences in astrocyte morphology in OVX + HE compared to free cycling HE in either branch points ([AUC]: 46.55 ± 4.08 OVX + HE vs. 55.92 ± 6.58 HE, two‐tailed unpaired Welch's *t*‐test *t*(30.44) = 1.211, *p* = 0.2352), process length ([AUC]: 1573 ± 159.7 OVX + HE vs. 1869 ± 207.4 HE, two‐tailed unpaired Welch's *t*‐test *t*(41.30) = 1.133, *p* = 0.2638), or process intersections ([AUC]: 233.6 ± 24.08 OVX + HE vs. 283.5 ± 31.38 HE, two‐tailed unpaired Welch's *t*‐test *t*(41.24) = 1.261, *p* = 0.2143). Similarly, there were no differences in astrocyte morphology on OVX + LE compared to free cycling LE in either branch points ([AUC]: 32.86 ± 6.26 OVX + LE vs. 20.25 ± 2.92 LE, two‐tailed unpaired Welch's *t*‐test *t*(28.26) = 1.824, *p* = 0.0787), process length ([AUC]: 758.8 ± 76.30 OVX + HE vs. 907.4 ± 186.9 HE, two‐tailed unpaired Welch's *t*‐test *t*(27.7) = 0.7361, *p* = 0.4679) or process intersections ([AUC]: 110.8 ± 11.19 OVX + LE vs. 130.0 ± 26.77 LE, two‐tailed unpaired Welch's *t*‐test *t*(28.08) = 0.6606, *p* = 0.5142) and results, therefore, were combined.

### 3.5 Estrogen Mediated Reduction in Caloric Intake Is Associated With Increased NMDA Receptor Activation in DMV Neurons

Previous studies have shown that caloric balance in response to acute high‐fat diet is dependent upon activation of astrocytes, release of astroglial transmitters, and the subsequent activation of extrasynaptic and synaptic NMDA receptors (NMDARex and NMDARs, respectively) (Clyburn et al. 2021, 2023). To determine whether activation of NMDARs is a common mechanism for homeostatic reductions in food intake, whole‐cell patch clamp recordings were made from DMV neurons in thin brainstem slices (300 μm) from LE and HE rats (*N* = 4, 7 respectively).

There was no difference in basal mEPSC properties between HE (*N* = 28) and LE (*N* = 18) DMV neurons. Briefly, mEPSC frequency was 2.62 ± 0.38 pulse per second in HE DMV neurons versus 2.77 ± 0.54 pulses per second in LE DMV neurons (two‐tailed unpaired Welch's *t*‐test *t*(33.67) = 0.2236, *p* = 0.8244), mEPSC amplitude was 27.86 ± 1.38 pA in HE DMV neurons versus 28.74 ± 1.67 pA in LE DMV neurons (two‐tailed unpaired Welch's *t*‐test, *t*(37.09) = 0.4079, *p* = 0.6857), while mEPSC charge transfer was 321.85 ± 62.74 pA.ms in HE DMV neurons versus 275.78 ± 82.40 in LE DMV neurons (two‐tailed unpaired Welch's *t*‐test *t*(35.01) = 0.4449, *p* = 0.6592) (see Table S1).

**FIGURE 3:**
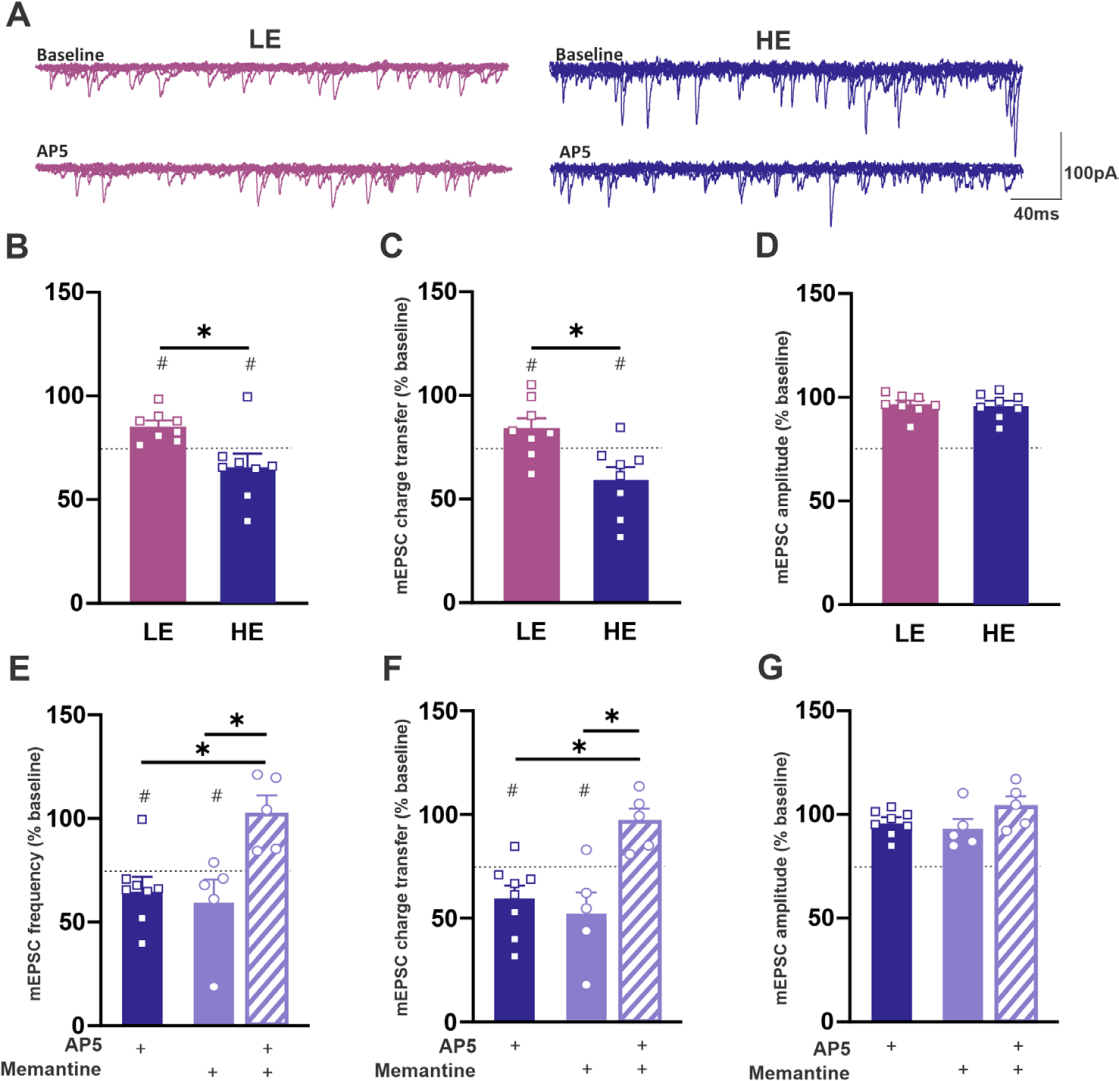
NMDARs are present in HE, but not LE, DMV neurons. (A) Representative mEPSC traces (6 overlapping consecutive traces) recorded from DMV neurons voltage clamped at −60 mV in LE and HE stages, respectively. Bath application of AP5 (25 μM) decreased mEPSC frequency, and charge transfer in 7/8 HE neurons whereas it had no effect on 0/8 LE neurons. Neurons were classified as responsive based on whether there was a > 25% reduction in frequency from baseline. (B–D) Graphical summary of the effects of AP5 on mEPSC frequency (B), charge transfer (C) and amplitude (D) in 8 LE neurons (*N* = 4 rats) and 8 HE neurons (*N* = 7 rats). (*p < 0.05, two‐tailed unpaired Welch's *t*‐test; ^#^ *p* < 0.05, two‐tailed one sample *t*‐test vs. baseline). (E–G) Graphical summary of the effects of AP5 subsequent to memantine on mEPSC frequency (E), charge transfer (F) and amplitude (G) in 5 HE neurons (*N* = 3 rats). Notably, application of memantine itself reduced mEPSC frequency and charge transfer in 4/5 neurons and no additional effect was observed with subsequent AP5 was application (0/5). All data are represented as mean ± SEM. (*p < 0.05, one‐way ANOVA with post hoc Bonferroni multiple comparison test; ^#^ *p* < 0.05, two‐tailed one sample *t*‐test vs. baseline).

In 8/8 LE DMV neurons, the NMDAs antagonist, AP5, induced a statistically‐significant, but physiologically non‐significant decrease (i.e., < 25% change; Clyburn et al. 2023; Carson et al. 2023) in mEPSC frequency (85.32% ± 2.58% of baseline, one sample *t*‐test *p* = 0.0007 (*μ* = 100%); Figure 3A,B) and charge transfer (84.04% ± 4.98% of baseline, one sample *t*‐test *p* = 0.0149 (*μ* = 100%); Figure 3C) but not amplitude (amplitude: 96.03% ± 2.18% of baseline, one sample *t*‐test *p* = 0.0927 (*μ* = 100%); Figure 3D). In contrast, AP5 decreased mEPSC frequency and charge transfer in 7/8 HE DMV neurons (frequency = 65.80% ± 6.03% of baseline, one sample *t*‐test *p* = 0.0008 (*μ* = 100%); Figure 3B, charge transfer = 59.60% ± 6.12% of baseline, one sample *t*‐test *p* = 0.0003 (*μ* = 100%); Figure 3C) but not amplitude (96.03% ± 2.18% of baseline, one sample *t*‐test *p* = 0.1104 [*m* = 100%]; Figure 3D). Furthermore, effects of AP5 on mEPSC frequency and charge transfer differed significantly between LE and HE (frequency: LE 85.32% ± 2.58% vs. HE 65.80% ± 6.03% of baseline; two‐tailed unpaired Welch's *t*‐test *t*(9.5) = 2.97, *p* = 0.0148; charge transfer: LE 84.04% ± 4.98% vs. HE 59.60% ± 6.12% of baseline; two‐tailed unpaired Welch's *t*‐test *t*(13.44) = 3.097, *p* = 0.0082; Figure 3B,C).

To determine whether the NMDA‐mediated currents observed in HE DMV neurons depend on activation of NMDARex, the ability of AP5 to modulate mEPSCs were assessed in the presence of the NMDARex antagonist memantine (30 μM). Memantine itself reduced mEPSC frequency (59.54% ± 10.58% of baseline, one‐sample *t*‐test *p* = 0.0187 (*μ* = 100%); Figure 3E) and charge transfer (52.34% ± 10.70% of baseline, one‐sample *t*‐test *p* = 0.0112 (*μ* = 100%); Figure 3F) in 4 out of 5 DMV neurons from three rats, while mEPSC amplitude remained unchanged (93.33% ± 4.54% of baseline, one‐sample *t*‐test *p* = 0.2160 (*μ* = 100%); Figure 3G). Subsequent application of AP5 in the presence of memantine had no additional effect on mEPSC properties (frequency: 102.92% ± 7.96% of baseline, one‐sample *t*‐test *p* = 0.7322 (*μ* = 100%); Figure 3E, charge transfer: 96.77% ± 6.12%, one‐sample *t*‐test *p* = 0.6256 (*μ* = 100%); Figure 3F, and amplitude: 104.0% ± 4.59%, one‐sample *t*‐test *p* = 0.4323 (*μ* = 100%); Figure 3G). When compared to the effects of AP5 alone, the effects of memantine + AP5 on mEPSC frequency (AP5: 65.80% ± 6.03% vs. Memantine+ AP5: 102.92% ± 7.96% of baseline, one‐way ANOVA, *F*(2, 17) = 7.794, *p* = 0.012, *post hoc* Bonferroni's multiple comparisons test, *p* = 0.0122; Figure 3E) and charge transfer (AP5: 59.60% ± 6.12% vs. memantine + AP5: 96.77% ± 6.12% of baseline, one‐way ANOVA, *F*(2, 17) = 8.651, *p* = 0.0032, post hoc Bonferroni's multiple comparisons test, *p* = 0.0092; Figure 3F) in HE DMV neurons were significantly different.

### 3.6 Blocking Estrogen Receptors Abolishes NMDARs Currents in HE Rats Whereas Activation of Estrogen Receptors Activates NMDARs in LE Rats

To further assess the role of estrogen in increasing glutamatergic drive to DMV neurons, the effects of AP5 were tested after bath application of the β‐estradiol receptor antagonist, ICI182,780 (10 μM), in HE rats, and following bath application of the estrogen receptor agonist, β‐estradiol (100 nM) in LE rats.

**FIGURE 4:**
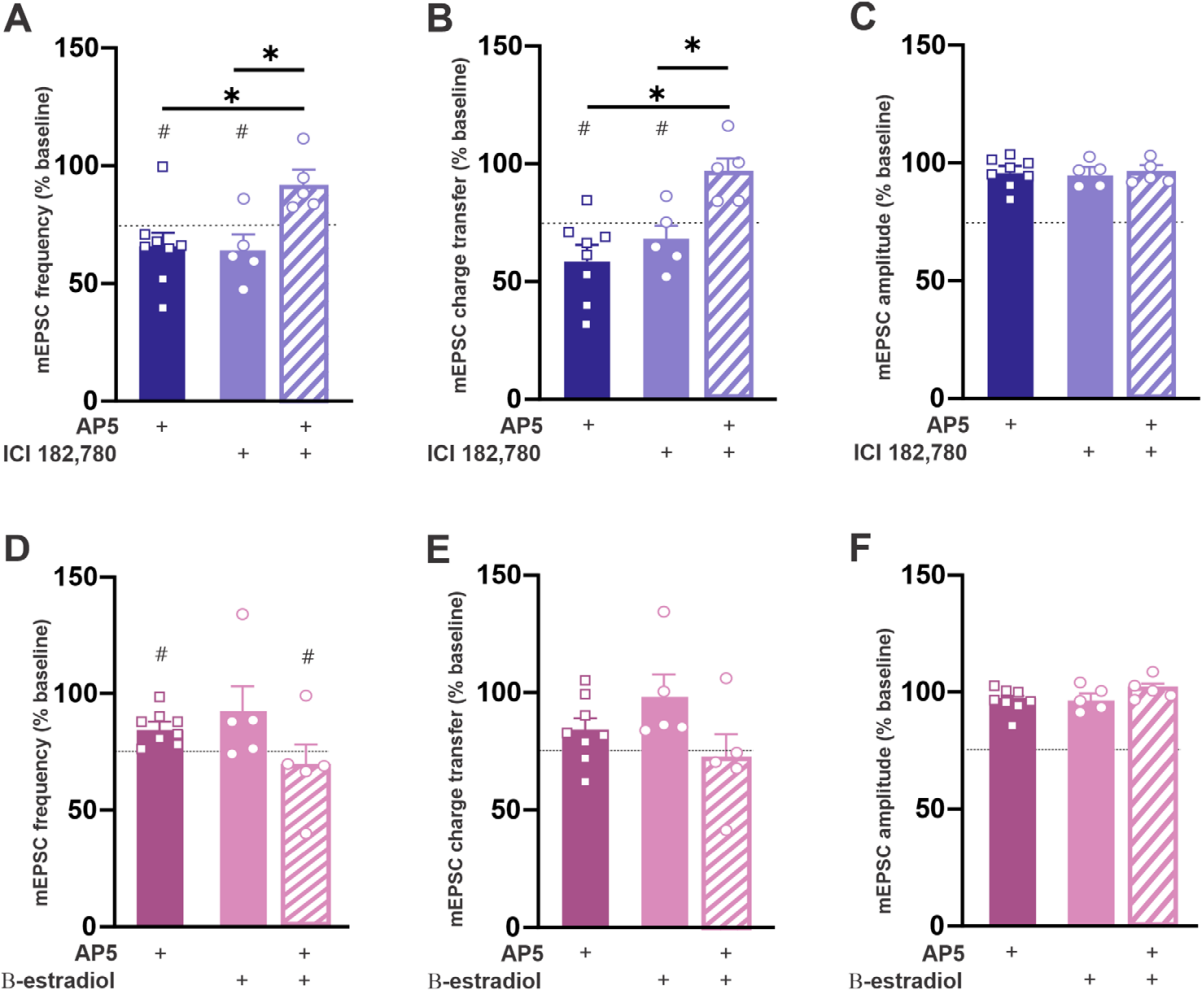
Blocking estrogen receptors abolishes NMDARs currents in HE rats, whereas activation of estrogen receptors activates NMDARs in LE rats. (A–C) Graphical summary of the effects of AP5 subsequent to estrogen receptor α antagonist, ICI182,780, on mEPSC frequency (A), charge transfer (B) and amplitude (C) in 5 HE neurons (*N* = 3 rats). Notably, application of ICI182,780 itself reduced mEPSC frequency and charge transfer in 4/5 neurons and no additional effect was observed with subsequent AP5 was application (0/5). (*p < 0.05, one‐way ANOVA with post hoc Bonferroni multiple comparison test; ^#^ *p* < 0.05, two‐tailed one sample *t*‐test vs. baseline). (D–F) Graphical summary of the effects of AP5 subsequent to Β estradiol, on mEPSC frequency (D), charge transfer (E), and amplitude (F) in 5 LE neurons (*N* = 3 rats). All data are represented as mean ± SEM. (^#^ *p* < 0.05, two‐tailed one sample *t*‐test vs. baseline).

Perfusion with the estrogen receptor antagonist, ICI182,780 decreased glutamatergic transmission in 5/5 HE DMV neurons (mEPSC frequency (64.50% ± 6.28%, one‐sample *t*‐test *p* = 0.0048 [*μ* = 100%])) and charge transfer (67.82% ± 5.92%, one‐sample *t*‐test *p* = 0.0056 [*μ* = 100%]) but not amplitude (95.56% ± 5.29%, one‐sample *t*‐test *p* = 0.1250 [*μ* = 100%]). Following application of ICI182,780, AP5 had no effect on glutamatergic transmission in any of these 5 HE DMV neurons, compared to the AP5 mediated decrease in 7/8 HE DMV neurons in the absence of ICI182,780; *χ* ^2^(1) = 9.479, *p* = 0.0021 (frequency: 92.61% ± 5.31%, one‐sample *t*‐test *p* = 0.2363 [*μ* = 100%]; charge transfer: 96.69% ± 5.93%, one‐sample *t*‐test *p* = 0.6063 [*μ* = 100%]; amplitude: 96.56% ± 2.12%, one‐sample *t*‐test *p* = 0.1806 [*μ* = 100%]; Figure 4A–C). That is, compared to the effect of AP5 alone, perfusion with ICI182,780 abolished the ability of AP5 to decrease mEPSC frequency and charge transfer. Specifically, the effects of AP5 on mEPSC frequency were reduced from 65.80% ± 6.03% of baseline to 92.61% ± 5.31% of baseline in the presence of ICI182,780 (one‐way ANOVA, *F*(2, 15) = 5.972, *p* = 0.0124, post hoc Bonferroni's multiple comparisons test, *p* = 0.0207), while the effects of AP5 on charge transfer were reduced from 59.6% ± 6.12% to 96.69% ± 5.93% of baseline in the presence of ICI182,780 (one‐way ANOVA, *F*(2, 15) = 9.340, *p* = 0.0023, post hoc Bonferroni's multiple comparisons test, *p* = 0.0021).

In contrast, in LE DMV neurons, the ability of the estrogen receptor agonist, β‐estradiol, to uncover AP5‐sensitive NMDARs currents was assessed. Although β‐estradiol alone had no effect on mEPSC properties (frequency: 92.23% ± 10.85% of baseline, one‐sample *t*‐test *p* = 0.5133 [*μ* = 100%]; charge transfer: 98.09% ± 2.41% of baseline, one‐sample *t*‐test *p* = 0.8516 [*μ* = 100%]; amplitude: 97.12% ± 2.32% of baseline, one‐sample *t*‐test *p* = 0.2807 [*μ* = 100%]), it uncovered the ability of AP5 to modulate mEPSCs in 4/5 LE DMV neurons (frequency: 68.89% ± 9.35% of baseline, one‐sample *t*‐test *p* = 0.0292 [*μ* = 100%]; charge transfer: 71.99% ± 10.31% of baseline, one‐sample *t*‐test *p* = 0.0221 [*μ* = 100%]), while amplitude remained unchanged (101.2% ± 1.8% of baseline, one‐sample *t*‐test *p* = 0.5253 [*μ* = 100%]; Figure 4D–F). Collectively, these data suggest that NMDARs‐dependent glutamatergic transmission to DMV neurons is dependent upon estrogen level.

### 3.7 Activation of Brainstem Astrocytes Induces a Decrease in Food Intake and Uncovers NMDARs Activity in DMV Neurons

**FIGURE 5:**
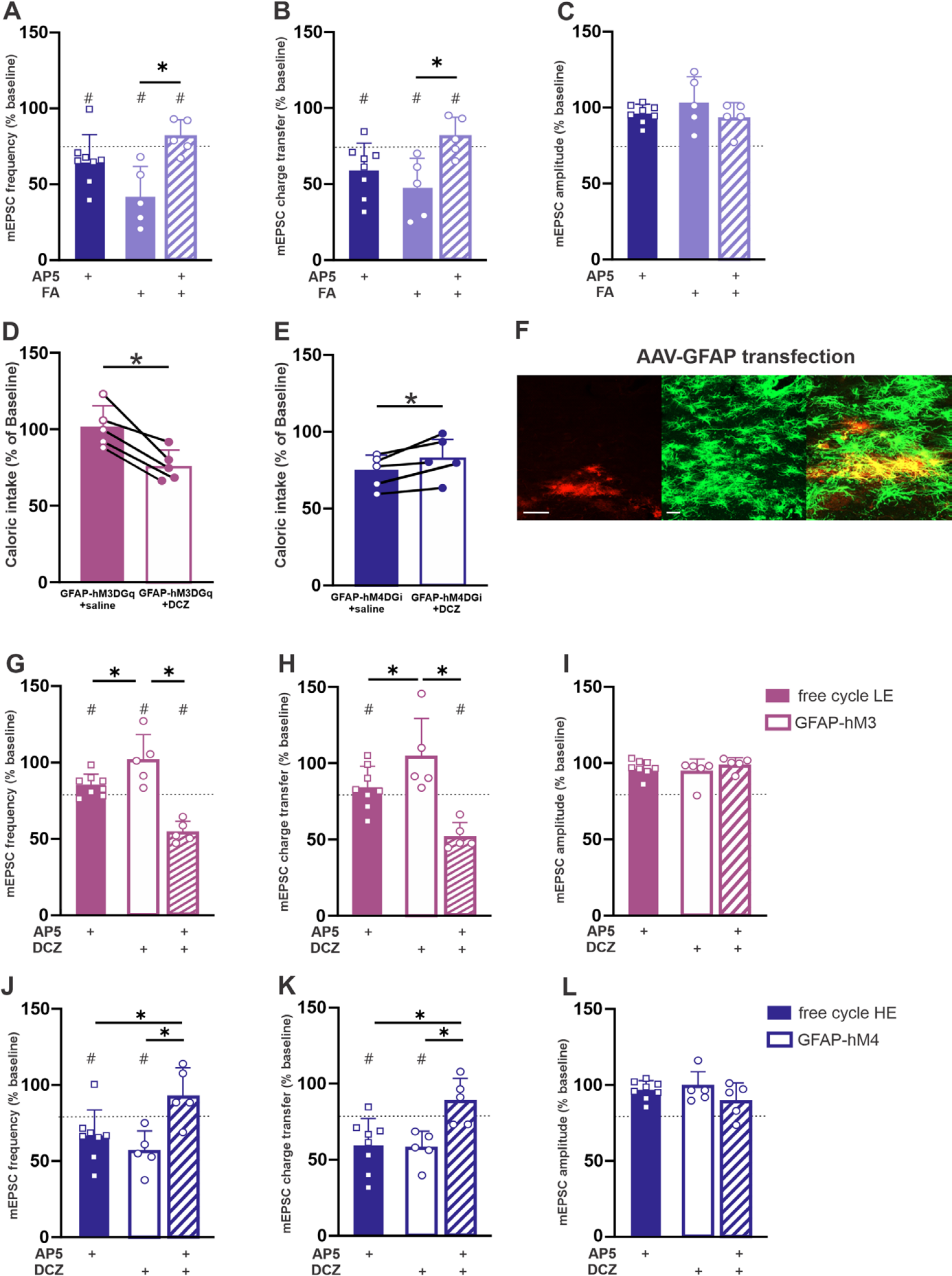
Stimulation of brainstem astrocytes decreases food intake and uncovers NMDARs activity in DMV neurons. (A–C) Graphical summary of the effects of AP5 after inhibition of astrocytes with fluoroacetate (FA), on mEPSC frequency (A), charge transfer (B) and amplitude (C) in 5 HE neurons (*N* = 3 rats). Note that FA itself reduced mEPSC frequency and charge transfer in 5/5 DMV neurons, while subsequent addition of AP5 had no further effect in 4/5 neurons. (*p < 0.05, one‐way ANOVA with post hoc Bonferroni multiple comparison test; ^#^ *p* < 0.05, two‐tailed one sample *t*‐test vs. baseline). (D) Graphical summary of caloric intake across 3 estrus cycles in LE before and after chemogenetic activation of astrocytes (GFAP‐hM3), expressed as percentage relative to the baseline. Activation of brainstem astrocytes reduced food intake independently from estrus stage. (E) Graphical summary of mean caloric intake across 3 estrus cycles in HE before and after chemogenetic inhibition of astrocytes (GFAP‐hM4), expressed as percentage relative to the baseline. Inhibition of brainstem astrocytes eliminated the cycle‐dependent oscillations in food intake. (*p < 0.05, two‐tailed paired Student's *t*‐test). (F) Micrograph illustrating post hoc identification of microinjection site and GFAP colocalization. (G–I) Graphical summary of the effects of AP5 after chemogenetic activation of astrocytes with DCZ, on mEPSC frequency (G), charge transfer (H) and amplitude (I) in 5 LE neurons (*N* = 3 rats). Note that activation of astrocytes uncovered the ability of AP5 to have an effect on mEPSC frequency and charge transfer in 5/5 LE DMV neurons compared to 0/8 in free cycling LE rats. (J–L) Graphical summary of the effects of AP5 after chemogenetic inhibition of astrocytes with DCZ, on mEPSC frequency (J), charge transfer (K) and amplitude (L) in 5 HE neurons (*N* = 3 rats). Note that inhibition of astrocytes blocked the ability of AP5 to reduce mEPSC frequency and charge transfer in 4/5 HE DMV neurons compared to 1/8 in free cycling HE rats. All data are represented as mean ± SEM. (*p < 0.05, one‐way ANOVA with post hoc Bonferroni multiple comparison test; ^#^ *p* < 0.05, two‐tailed one sample *t*‐test vs. baseline).

To assess whether estrogen‐dependent reduction in food intake occurs due to activated brainstem astrocytes and subsequent activation of NMDARs on DMV neurons, the effect of AP5 on glutamatergic transmission was assessed following pharmacological inhibition of astrocytes with fluoroacetate (FA; 100 μM). In HE rats, bath application of FA led to a significant reduction in mEPSC frequency and charge transfer (frequency: 42.2% ± 8.81% of baseline, one‐sample *t*‐test *p* = 0.0028 [*μ* = 100%]; charge transfer: 47.27% ± 8.79% of baseline, one‐sample *t*‐test *p* = 0.0039 [*μ* = 100%]), while the amplitude remained unchanged (103.3% ± 7.60% of baseline, one‐sample *t*‐test *p* = 0.6845 [*μ* = 100%]; Figure 5A–C). In the presence of FA, subsequent addition of AP5 induced a statistically significant, but physiologically nonsignificant (i.e., < 25% change) decrease in mEPSC frequency in 1 of 5 DMV neurons (compared to 7/8 HE DMV neurons in the absence of FA; *χ* ^2^(1) = 5.923, *p* = 0.0149 compared to AP5 alone; frequency: 81.34% ± 5.00% of baseline, one‐sample *t*‐test *p* = 0.02 [*μ* = 100%]) and charge transfer (81.23% ± 5.76% of baseline, one‐sample *t*‐test *p* = 0.0310 [*μ* = 100%]). A significant difference was observed in the effect of FA alone on glutamatergic transmission compared to FA + AP5 (frequency: FA 42.2% ± 8.81% vs. 81.34% ± 5.00% of baseline, one‐way ANOVA, *F*(2, 15) = 7.174, *p* = 0.0065, post hoc Bonferroni's multiple comparisons test, *p* = 0.0051; charge transfer: 47.27% ± 8.79% vs. 81.23% ± 5.76% of baseline, one‐way ANOVA, *F*(2, 15) = 5.185, *p* = 0.0194, post hoc Bonferroni's multiple comparisons test, *p* = 0.0165; Figure 5A,B).

To further investigate the interplay between estrogen and brainstem astrocytes in modulating food intake, in five rats DMV astrocytes were transfected with pAAV‐GFAP‐hM3D(Gq)‐mCherry (“GFAP‐hM3”), while another five rats were transfected with pAAV‐GFAP‐hM4D(Gi)‐mCherry (“GFAP‐hM4”). Administration of the DREADD agonist, DCZ (0.1 mg/kg i.p.) to GFAP‐hM3 animals led to a significant reduction in food intake while in the LE state (LE control 101.7 ± 6.2 vs. LE DCZ 76.2 ± 4.6 kcal/g, two‐tailed paired Student's *t*‐test *t*(4) = 5.350, *p* = 0.0059; Figure 5D). In contrast, chemogenetic inhibition of astrocytes prevented the HE‐dependent decrease in caloric intake (HE control 73.8 ± 4.8 vs. HE DCZ 83.0 ± 6.2 kcal/g, two‐tailed paired Student's *t*‐test *t*(4) = 3.104, *p* = 0.0361; Figure 5E).

To confirm that estrogen activates brainstem astrocytes to induce NMDARs‐currents in DMV neurons, whole cell patch clamp recordings were made from DMV neurons near mCherry‐labeled astrocytes in GFAP‐hM3 or GFAP‐hM4 transfected brainstems. In contrast to LE DMV neurons where AP5 had no effect on mEPSC properties in 8/8 neurons, in LE GFAP‐hM3 rats, DCZ uncovered the ability of AP5 to decrease mEPSC frequency and charge transfer in 5/5 neurons (frequency: DCZ 101.76% ± 7.43% vs. DCZ + AP5 54.61% ± 3.09% of baseline, one‐way ANOVA, *F*(2, 15) = 14.03, *p* = 0.0004, post hoc Bonferroni's multiple comparisons test, *p* = 0.0004; charge transfer: DCZ 104.28% ± 11.21% vs. DCZ + AP5 52.36% ± 3.98% of baseline, one‐way ANOVA, *F*(2, 15) = 12.63, *p* = 0.0006, post hoc Bonferroni's multiple comparisons test, *p* = 0.0011; *χ* ^2^(1) = 13.00, *p* = 0.0003 compared to LE + AP5 alone; Figure 5G,H).

Conversely, in HE DMV neurons, bath application of DCZ itself decreased mEPSC frequency and charge transfer (frequency: 56.28% ± 6.07% of baseline, one‐sample *t*‐test *p* = 0.0020 [*μ* = 100%]; Figure 5J; charge transfer: 57.62% ± 5.02% of baseline, one‐sample *t*‐test *p* = 0.0011, [*μ* = 100%]; Figure 5K) in 4/5 neurons, and abolished the ability of AP5 to decrease glutamatergic transmission. Specifically, in contrast to the effects of AP5 to decrease mEPSCs in 7/8 HE neurons, in GFAP‐hM4 rats in the presence of DCZ, AP5 had no effect in 4/5 neurons (frequency: DCZ 56.28% ± 6.07% vs. DCZ + AP5 93.51% ± 7.99% of baseline, one‐way ANOVA, *F*(2, 15) = 12.40, *p* = 0.0007, post hoc Bonferroni's multiple comparisons test, *p* = 0.0008; charge transfer: DCZ 57.62% ± 5.01% vs. DCZ + AP5 88.36% ± 6.76% of baseline, one‐way ANOVA, *F*(2, 15) = 7.771, *p* = 0.0048, post hoc Bonferroni's multiple comparisons test, *p* = 0.0076; *χ* ^2^(1) = 5.923, *p* = 0.0149 compared to HE + AP5 alone Figure 5J,K).

Together, these data suggest that inhibiting brainstem astrocytes in HE states attenuates NMDAR‐mediated signaling in DMV neurons, while activating astrocytes uncovers NMDARs‐mediated currents in LE states and, further, that these astrocyte‐dependent effects on glutamatergic transmission are responsible for estrogen‐dependent effects on caloric intake.

## 4 Discussion

The results from the current study suggest that increased estrogen levels (i) activate brainstem astrocytes, which, in turn, (ii) activate NMDARex and subsequently NMDARs receptors on DMV neurons, which (iii) are responsible for the physiological oscillation in caloric intake observed in female rats.

Although fluctuations in food intake across the estrus cycle have been well described (Asarian and Geary 2006; Drewett 1973; Eckel et al. 2000; Tarttelin and Gorski 1971; Wade 1975), the mechanistic basis of this effect remains incompletely understood. The decrease in food intake during high estrogen states has often been attributed to hypothalamic‐dependent actions (Campos et al. 2013; Campos and Ritter 2015; Ritter 2011; Santollo et al. 2013) while the present study demonstrates that brainstem vagal circuits also contribute to this homeostatic regulation of caloric balance.

DMV neurons are spontaneously active pacemaker cells, whose firing rate and excitability are regulated by GABAergic, glutamatergic, and catecholaminergic synaptic inputs from the NTS (Travagli et al. 1991; Davis et al. 2004; Rogers et al. 2003). Under physiological conditions, both in vivo and in vitro studies have demonstrated that inhibitory GABAergic inputs tonically regulate the activity of DMV neurons, whereas excitatory glutamatergic inputs do not appear to play a significant role in regulating DMV neuronal activity under basal conditions (Travagli et al. 1991; Babic et al. 2012; Sivarao et al. 1998). Glutamatergic signaling at the NTS‐DMV synapse is mediated via AMPA receptors, most likely due to the voltage‐dependent Mg^2+^ block of the NMDA ion channel pore at the resting membrane potential (Travagli et al. 1991; Broussard et al. 1997). Despite this, previous studies have implicated hindbrain NMDA receptor signaling in satiation and meal‐size control (Campos and Ritter 2015; Ritter 2011; Wright et al. 2011; Treece et al. 1998).

We have previously shown that exposure to an acute high‐fat diet triggers an astrocyte‐dependent activation of extrasynaptic NMDARs, leading to an increase in glutamatergic signaling to DMV neurons, which ultimately restores caloric balance (Clyburn et al. 2019, 2021, 2023). The present study extended the role of DVC astrocytes in homeostatic regulation of caloric balance to include their involvement in estrus cycle‐dependent oscillations in food intake. Chemogenetic inhibition of astrocytes eliminated the observed fluctuations in caloric intake, resulting in a sustained elevated intake, whereas chemogenetic activation of astrocytes was sufficient to reduce caloric intake regardless of the estrus stage. These results indicate both the necessity and sufficiency of brainstem astrocytes in regulating food intake across the estrus cycle.

Estrogen receptors are expressed on astrocytes (Azcoitia et al. 1999; Kuo et al. 2010), microglia (Tapia‐Gonzalez et al. 2008; Liu et al. 2005), and neurons, including those within the DVC (Vanderhorst et al. 2009; Simonian et al. 1998; Chi et al. 2011) express estrogen receptors and are likely to respond directly to circulating estrogen, particularly within the DVC, which lies adjacent to circumventricular regions lacking a fully intact blood–brain barrier (Borison 1989; Wang et al. 2008). The present study demonstrated that blocking estrogen signaling via application of an estrogen receptor antagonist eliminated the increase in glutamatergic drive onto DMV neurons observed in high estrogen states. A similar loss of glutamatergic NMDARs signaling was observed following pharmacologic or chemogenetic astrocyte inhibition; however, it should be noted that pharmacological inhibition was less effective than chemogenetic inhibition which might suggest that the concentration of FA and/or the duration of the drug application may not be sufficient to fully inhibit all the astrocytic activity. Furthermore, inhibition of extrasynaptic NMDAR also resulted in a loss of HE‐dependent NMDARs signaling, suggesting that estrogen signaling, astrocyte adaptation, and a glutamatergic signaling cascade work in concert to regulate DMV neuronal activity. While the present study did not investigate the site at which estrogen acts to induce the observed responses (astrocytes vs. microglia vs. neurons) or the mechanistic sequence of extrasynaptic versus synaptic NMDAR activation, our previous studies have shown that astrocytic release of gliotransmitters ATP and glutamate leads to activation of group 1 metabotropic glutamate receptors and purinergic P2X receptors on DMV neurons, respectively, which have been identified as a critical component of the postsynaptic signaling cascade (Clyburn et al. 2023; Jourdain et al. 2007; Clyburn and Browning 2019) that results in activation of extrasynaptic and then synaptic NMDAR. It is therefore possible that estrogen‐dependent adaptations in DVC astrocytes engage a similar mechanism to activate extrasynaptic NMDA receptor signaling in the present study.

The present immunohistochemical studies indicated that alterations in DVC astrocytes occur during periods of high estrus, with evidence of changes in astrocyte morphological complexity, suggestive of a mild, non‐proliferative astrocytic state rather than classical reactive astrogliosis (Verkhratsky et al. 2023). Such changes may allow the rapid astrocyte‐dependent modulation of synaptic signaling without structural remodeling, which could be well‐suited to rapid activation and deactivation across the estrus cycle. Consistent with this, the chemogenetic modulation of astrocytes was sufficient to abolish the estrus‐dependent oscillations in food intake and caloric balance. Together, these results suggest that brainstem astrocytes are involved in the estrus‐dependent oscillation in food intake.

In summary, the present study suggests that estrogen‐dependent modulation of DVC astrocytes upregulates NMDA receptor signaling in DMV neurons, thereby contributing to physiological oscillations in food intake across the estrus cycle. While the repetitive and cyclical nature of the observed astrocyte alterations presumably reflects physiological adaptations rather than reactive astrogliosis, further studies will be required to determine the exact nature of the changes in astrocyte phenotype. Results of the current study may provide potential mechanistic insights into how altered estrogen signaling may impact food intake and energy balance.

## Author Contributions

**K. Selin Ozkaya:** conceptualization, investigation, writing – original draft, methodology, validation, writing – review and editing, formal analysis, data curation. **Ceyda Yalcin:** writing – original draft, methodology, writing – review and editing. **Ruchi Bhagat:** methodology, writing – review and editing. **Emily E. Haar:** methodology, writing – review and editing. **Kirsteen N. Browning:** conceptualization, investigation, funding acquisition, writing – original draft, methodology, writing – review and editing, project administration, formal analysis, data curation, supervision, resources.

## Funding

The authors wish to thank NIH grants NIDDK 120170 and DK 111667 for their support.

## Conflicts of Interest

The authors declare no conflicts of interest.

## Supporting information


**Table S1:** mEPSC properties.
**Figure S1:** Serum estradiol measurements.

## Data Availability

All data will be made available by the corresponding author upon request.

## References

[jnc70498-bib-0001] Asarian, L. , and N. Geary . 2006. “Modulation of Appetite by Gonadal Steroid Hormones.” Philosophical Transactions of the Royal Society of London. Series B, Biological Sciences 361, no. 1471: 1251–1263. 10.1098/rstb.2006.1860. 16815802 PMC1642706

[jnc70498-bib-0002] Azcoitia, I. , A. Sierra , and L. M. Garcia‐Segura . 1999. “Localization of Estrogen Receptor Beta‐Immunoreactivity in Astrocytes of the Adult Rat Brain.” Glia 26, no. 3: 260–267. 10340766

[jnc70498-bib-0003] Babic, T. , K. N. Browning , Y. Kawaguchi , X. Tang , and R. A. Travagli . 2012. “Pancreatic Insulin and Exocrine Secretion Are Under the Modulatory Control of Distinct Subpopulations of Vagal Motoneurones in the Rat.” Journal of Physiology 590, no. 15: 3611–3622. 10.1113/jphysiol.2012.234955. 22711959 PMC3547274

[jnc70498-bib-0004] Blaustein, J. D. , and G. N. Wade . 1976. “Ovarian Influences on the Meal Patterns of Female Rats.” Physiology & Behavior 17, no. 2: 201–208. 10.1016/0031-9384(76)90064-0. 1033580

[jnc70498-bib-0005] Borison, H. L. 1989. “Area Postrema: Chemoreceptor Circumventricular Organ of the Medulla Oblongata.” Progress in Neurobiology 32, no. 5: 351–390. 10.1016/0301-0082(89)90028-2. 2660187

[jnc70498-bib-0006] Broussard, D. L. , H. Li , and S. M. Altschuler . 1997. “Colocalization of GABA(A) and NMDA Receptors Within the Dorsal Motor Nucleus of the Vagus Nerve (DMV) of the Rat.” Brain Research 763, no. 1: 123–126. 10.1016/s0006-8993(97)00344-2. 9272836

[jnc70498-bib-0007] Buffenstein, R. , S. D. Poppitt , R. M. McDevitt , and A. M. Prentice . 1995. “Food Intake and the Menstrual Cycle: A Retrospective Analysis, With Implications for Appetite Research.” Physiology & Behavior 58, no. 6: 1067–1077. 10.1016/0031-9384(95)02003-9. 8623004

[jnc70498-bib-0008] Butcher, R. L. , W. E. Collins , and N. W. Fugo . 1974. “Plasma Concentration of LH, FSH, Prolactin, Progesterone and Estradiol‐17beta Throughout the 4‐Day Estrous Cycle of the Rat.” Endocrinology 94, no. 6: 1704–1708. 10.1210/endo-94-6-1704. 4857496

[jnc70498-bib-0009] Campos, C. A. , and R. C. Ritter . 2015. “NMDA‐Type Glutamate Receptors Participate in Reduction of Food Intake Following Hindbrain Melanocortin Receptor Activation.” American Journal of Physiology. Regulatory, Integrative and Comparative Physiology 308, no. 1: R1–R9. 10.1152/ajpregu.00388.2014. 25394828 PMC4281681

[jnc70498-bib-0010] Campos, C. A. , H. Shiina , M. Silvas , S. Page , and R. C. Ritter . 2013. “Vagal Afferent NMDA Receptors Modulate CCK‐Induced Reduction of Food Intake Through Synapsin I Phosphorylation in Adult Male Rats.” Endocrinology 154, no. 8: 2613–2625. 10.1210/en.2013-1062. 23715865 PMC3713210

[jnc70498-bib-0011] Carson, K. E. , J. Alvarez , J. Q. Mackley , R. A. Travagli , and K. N. Browning . 2023. “Perinatal High‐Fat Diet Exposure Alters Oxytocin and Corticotropin Releasing Factor Inputs Onto Vagal Neurocircuits Controlling Gastric Motility.” Journal of Physiology 601, no. 14: 2853–2875. 10.1113/JP284726. 37154244 PMC10524104

[jnc70498-bib-0012] Chi, J. H. , K. Narita , T. Ichimaru , and T. Murata . 2011. “Estrogen Increases c‐Fos Expression in the Paraventricular Nucleus Along With Its Anorexic Effect in Developing Rats.” Journal of Reproduction and Development 57, no. 3: 365–372. 10.1262/jrd.10-189e. 21358146

[jnc70498-bib-0013] Clyburn, C. , and K. N. Browning . 2019. “Role of Astroglia in Diet‐Induced Central Neuroplasticity.” Journal of Neurophysiology 121, no. 4: 1195–1206. 10.1152/jn.00823.2018. 30699056 PMC6485736

[jnc70498-bib-0014] Clyburn, C. , K. E. Carson , C. R. Smith , R. A. Travagli , and K. N. Browning . 2023. “Brainstem Astrocytes Control Homeostatic Regulation of Caloric Intake.” Journal of Physiology 601, no. 4: 801–829. 10.1113/JP283566. 36696965 PMC10026361

[jnc70498-bib-0015] Clyburn, C. , C. A. Howe , A. C. Arnold , C. H. Lang , R. A. Travagli , and K. N. Browning . 2019. “Perinatal High‐Fat Diet Alters Development of GABA(A) Receptor Subunits in Dorsal Motor Nucleus of Vagus.” American Journal of Physiology. Gastrointestinal and Liver Physiology 317, no. 1: G40–G50. 10.1152/ajpgi.00079.2019. 31042399 PMC6689732

[jnc70498-bib-0016] Clyburn, C. , R. A. Travagli , A. C. Arnold , and K. N. Browning . 2021. “DMV Extrasynaptic NMDA Receptors Regulate Caloric Intake in Rats.” JCI Insight 6, no. 9: e139785. 10.1172/jci.insight.139785. 33764905 PMC8262316

[jnc70498-bib-0017] Davis, S. F. , A. V. Derbenev , K. W. Williams , N. R. Glatzer , and B. N. Smith . 2004. “Excitatory and Inhibitory Local Circuit Input to the Rat Dorsal Motor Nucleus of the Vagus Originating From the Nucleus Tractus Solitarius.” Brain Research 1017, no. 1–2: 208–217. 10.1016/j.brainres.2004.05.049. 15261116 PMC3761086

[jnc70498-bib-0018] Drewett, R. F. 1973. “Oestrous and Dioestrous Components of the Ovarian Inhibition on Hunger in the Rat.” Animal Behaviour 21, no. 4: 772–780. 10.1016/s0003-3472(73)80103-4. 4798198

[jnc70498-bib-0019] Drewett, R. F. 1974. “The Meal Patterns of the Oestrous Cycle and Their Motivational Significance.” Quarterly Journal of Experimental Psychology 26, no. Pt3: 489–494. 10.1080/14640747408400438. 4472372

[jnc70498-bib-0020] Eckel, L. A. 2004. “Estradiol: A Rhythmic, Inhibitory, Indirect Control of Meal Size.” Physiology & Behavior 82, no. 1: 35–41. 10.1016/j.physbeh.2004.04.023. 15234587

[jnc70498-bib-0021] Eckel, L. A. , T. A. Houpt , and N. Geary . 2000. “Spontaneous Meal Patterns in Female Rats With and Without Access to Running Wheels.” Physiology & Behavior 70, no. 3–4: 397–405. 10.1016/s0031-9384(00)00278-x. 11006440

[jnc70498-bib-0022] Gong, E. J. , D. Garrel , and D. H. Calloway . 1989. “Menstrual Cycle and Voluntary Food Intake.” American Journal of Clinical Nutrition 49, no. 2: 252–258. 10.1093/ajcn/49.2.252. 2916445

[jnc70498-bib-0023] Jourdain, P. , L. H. Bergersen , K. Bhaukaurally , et al. 2007. “Glutamate Exocytosis From Astrocytes Controls Synaptic Strength.” Nature Neuroscience 10, no. 3: 331–339. 10.1038/nn1849. 17310248

[jnc70498-bib-0024] Kuo, J. , N. Hamid , G. Bondar , P. Dewing , J. Clarkson , and P. Micevych . 2010. “Sex Differences in Hypothalamic Astrocyte Response to Estradiol Stimulation.” Biology of Sex Differences 1, no. 1: 7. 10.1186/2042-6410-1-7. 21208471 PMC3016240

[jnc70498-bib-0025] Liu, X. , X. L. Fan , Y. Zhao , et al. 2005. “Estrogen Provides Neuroprotection Against Activated Microglia‐Induced Dopaminergic Neuronal Injury Through Both Estrogen Receptor‐Alpha and Estrogen Receptor‐Beta in Microglia.” Journal of Neuroscience Research 81, no. 5: 653–665. 10.1002/jnr.20583. 16013043

[jnc70498-bib-0026] López, M. , and M. Tena‐Sempere . 2015. “Estrogens and the Control of Energy Homeostasis: A Brain Perspective.” Trends in Endocrinology and Metabolism 26, no. 8: 411–421. 10.1016/j.tem.2015.06.003. 26126705

[jnc70498-bib-0027] Lyons, P. M. , A. S. Truswell , M. Mira , J. Vizzard , and S. F. Abraham . 1989. “Reduction of Food Intake in the Ovulatory Phase of the Menstrual Cycle.” American Journal of Clinical Nutrition 49, no. 6: 1164–1168. 10.1093/ajcn/49.6.1164. 2729155

[jnc70498-bib-0028] Martínez de Morentin Pablo, B. , I. González‐García , L. Martins , et al. 2014. “Estradiol Regulates Brown Adipose Tissue Thermogenesis via Hypothalamic AMPK.” Cell Metabolism 20, no. 1: 41–53. 10.1016/j.cmet.2014.03.031. 24856932 PMC4082097

[jnc70498-bib-0029] Mauvais‐Jarvis, F. , D. J. Clegg , and A. L. Hevener . 2013. “The Role of Estrogens in Control of Energy Balance and Glucose Homeostasis.” Endocrine Reviews 34, no. 3: 309. 10.1210/er.2012-1055. 23460719 PMC3660717

[jnc70498-bib-0030] Ritter, R. C. 2011. “A Tale of Two Endings: Modulation of Satiation by NMDA Receptors on or Near Central and Peripheral Vagal Afferent Terminals.” Physiology & Behavior 105, no. 1: 94–99. 10.1016/j.physbeh.2011.02.042. 21382391 PMC3181280

[jnc70498-bib-0031] Rogers, R. C. , R. A. Travagli , and G. E. Hermann . 2003. “Noradrenergic Neurons in the Rat Solitary Nucleus Participate in the Esophageal‐Gastric Relaxation Reflex.” American Journal of Physiology. Regulatory, Integrative and Comparative Physiology 285, no. 2: R479–R489. 10.1152/ajpregu.00155.2003. 12714355 PMC3062485

[jnc70498-bib-0032] Santollo, J. , A. Marshall , and D. Daniels . 2013. “Activation of Membrane‐Associated Estrogen Receptors Decreases Food and Water Intake in Ovariectomized Rats.” Endocrinology 154, no. 1: 320–329. 10.1210/en.2012-1858. 23183173 PMC3529383

[jnc70498-bib-0033] Simonian, S. X. , B. Delaleu , A. Caraty , and A. E. Herbison . 1998. “Estrogen Receptor Expression in Brainstem Noradrenergic Neurons of the Sheep.” Neuroendocrinology 67, no. 6: 392–402. 10.1159/000054338. 9662719

[jnc70498-bib-0034] Sivarao, D. V. , Z. K. Krowicki , and P. J. Hornby . 1998. “Role of GABAA Receptors in Rat Hindbrain Nuclei Controlling Gastric Motor Function.” Neurogastroenterology and Motility 10, no. 4: 305–313. 10.1046/j.1365-2982.1998.00110.x. 9697105

[jnc70498-bib-0035] Strom, J. O. , A. Theodorsson , E. Ingberg , I. M. Isaksson , and E. Theodorsson . 2012. “Ovariectomy and 17beta‐Estradiol Replacement in Rats and Mice: A Visual Demonstration.” Journal of Visualized Experiments 64, no. 64: e4013. 10.3791/4013. PMC3471296 22710371

[jnc70498-bib-0036] Tapia‐Gonzalez, S. , P. Carrero , O. Pernia , L. M. Garcia‐Segura , and Y. Diz‐Chaves . 2008. “Selective Oestrogen Receptor (ER) Modulators Reduce Microglia Reactivity In Vivo After Peripheral Inflammation: Potential Role of Microglial ERs.” Journal of Endocrinology 198, no. 1: 219–230. 10.1677/JOE-07-0294. 18460549

[jnc70498-bib-0037] Tarttelin, M. F. , and R. A. Gorski . 1971. “Variations in Food and Water Intake in the Normal and Acyclic Female Rat.” Physiology & Behavior 7, no. 6: 847–852. 10.1016/0031-9384(71)90050-3. 5167385

[jnc70498-bib-0038] ter Haar, M. B. 1972. “Circadian and Estrual Rhythms in Food Intake in the Rat.” Hormones and Behavior 3, no. 3: 213–219. 10.1016/0018-506x(72)90034-7. 4681745

[jnc70498-bib-0039] Travagli, R. A. , R. A. Gillis , C. D. Rossiter , and S. Vicini . 1991. “Glutamate and GABA‐Mediated Synaptic Currents in Neurons of the Rat Dorsal Motor Nucleus of the Vagus.” American Journal of Physiology. Gastrointestinal and Liver Physiology 260, no. 3: G531–G536. 10.1152/ajpgi.1991.260.3.G531. 1672243

[jnc70498-bib-0040] Treece, B. R. , M. Covasa , R. C. Ritter , and G. A. Burns . 1998. “Delay in Meal Termination Follows Blockade of N‐Methyl‐D‐Aspartate Receptors in the Dorsal Hindbrain.” Brain Research 810, no. 1–2: 34–40. 10.1016/s0006-8993(98)00867-1. 9813231

[jnc70498-bib-0041] Vanderhorst, V. G. , E. Terasawa , and H. J. Ralston 3rd . 2009. “Estrogen Receptor‐Alpha Immunoreactive Neurons in the Brainstem and Spinal Cord of the Female Rhesus Monkey: Species‐Specific Characteristics.” Neuroscience 158, no. 2: 798–810. 10.1016/j.neuroscience.2008.10.017. 18996446 PMC4641676

[jnc70498-bib-0042] Verkhratsky, A. , A. Butt , B. Li , et al. 2023. “Astrocytes in Human Central Nervous System Diseases: A Frontier for New Therapies.” Signal Transduction and Targeted Therapy 8, no. 1: 396. 10.1038/s41392-023-01628-9. 37828019 PMC10570367

[jnc70498-bib-0043] Wade, G. N. 1975. “Some Effects of Ovarian Hormones on Food Intake and Body Weight in Female Rats.” Journal of Comparative and Physiological Psychology 88, no. 1: 183–193. 10.1037/h0076186. 1120795

[jnc70498-bib-0044] Wallen, W. J. , M. P. Belanger , and C. Wittnich . 2001. “Sex Hormones and the Selective Estrogen Receptor Modulator Tamoxifen Modulate Weekly Body Weights and Food Intakes in Adolescent and Adult Rats.” Journal of Nutrition 131, no. 9: 2351–2357. 10.1093/jn/131.9.2351. 11533278

[jnc70498-bib-0045] Wang, Q. P. , J. L. Guan , W. Pan , A. J. Kastin , and S. Shioda . 2008. “A Diffusion Barrier Between the Area Postrema and Nucleus Tractus Solitarius.” Neurochemical Research 33, no. 10: 2035–2043. 10.1007/s11064-008-9676-y. 18373195

[jnc70498-bib-0046] Wright, J. , C. Campos , T. Herzog , M. Covasa , K. Czaja , and R. C. Ritter . 2011. “Reduction of Food Intake by Cholecystokinin Requires Activation of Hindbrain NMDA‐Type Glutamate Receptors.” American Journal of Physiology. Regulatory, Integrative and Comparative Physiology 301, no. 2: R448–R455. 10.1152/ajpregu.00026.2011. 21562094 PMC3154714

