# Supplementary material for: Brainstem Astrocytes Regulate Estrus‐Dependent Oscillations in Food Intake
Source: J Neurochem. 2026 Jun 18;170(6):e70498. doi: 10.1111/jnc.70498 (PMC13277959; doi:10.1111/jnc.70498)
Supplement: Supplementary file 1 — Table S1: mEPSC properties. Figure S1: Serum estradiol measurements. [file JNC-170-e70498-s001.docx]

**Supplementary Table 1: mEPSC properties**

| **Condition** | **Treatment** | **mEPSC Measurement** | | |
| --- | --- | --- | --- | --- |
|  |  | **Frequency**  **(pulse per second)** | **Charge Transfer**  **(pA.ms)** | **Amplitude**  **(pA)** |
| **LE** | **Baseline** | **2.75±0.52** | **275.78±82.39** | **28.74±1.67** |
|  | Baseline  + AP5 | 3.85±1.04  3.20±0.83 | 461.70±141.22  369.35±105.12 | 31.84±2.39  30.76±2.52 |
|  | Baseline  + β estradiol  + β estradiol + AP5 | 1.94±0.73  1.64±0.52  1.11±0.36 | 251.10±124.27  225.57±100.43  157.20±67.09 | 26.27±2.27  25.48±2.18  25.75±1.90 |
|  | Baseline (GFAP-hM3’)  + DCZ  + DCZ+AP5 | 1.89±0.40  1.85±0.36  **1.00±0.20^*^** | 173.03±36.03  171.19±33.06  **88.88±17.07^*^** | 26.25±3.85  24.20±2.63  24.10±2.86 |
| **HE** | **Baseline** | **3.04±0.57** | **321.85±62.74** | **27.86±1.38** |
|  | Baseline  + AP5 | 4.41±0.77  **3.11±0.86****^*^** | 628.12±149.91  **404.14±140.83^*^** | 32.22±3.97  30.62±3.34 |
|  | Baseline  + Memantine  + Memantine + AP5 | 1.60±0.29  0.86±0.16  0.92±0.21 | 152.88±39.99  **72.18±20.55**  **72.70±22.40** | 25.64±1.46  24.01±2.11  24.84±2.00 |
|  | Baseline  + ICI,182 780  + ICI,182 780 + AP5 | 1.64±0.36  **1.01±0.22**  **0.91±0.17** | 164.86±39.62  1**04.51±22.97**  **96.69±5.93** | 25.58±1.65  24.43±1.69  23.58±1.72 |
|  | Baseline  + FA  + FA+AP5 | 1.86±0.64  0.64±0.19  0.50±0.14 | 213.57±107.76  74.46±28.15  63.57±28.12 | 28.44±2.29  29.12±2.46  27.31±2.88 |
|  | Baseline (GFAP-hM4’)  + DCZ  + DCZ+AP5 | 2.54±1.30  1.22±0.46  1.15±0.42 | 266.06±128.61  132.08±48.69  120.39±45.85 | 24.78±2.21  24.66±3.42  21.56±1.62 |

*****p<0.05 vs baseline (Student’s paired t-test)

**Supplementary Figure 1: Serum Estradiol Measurements**

**
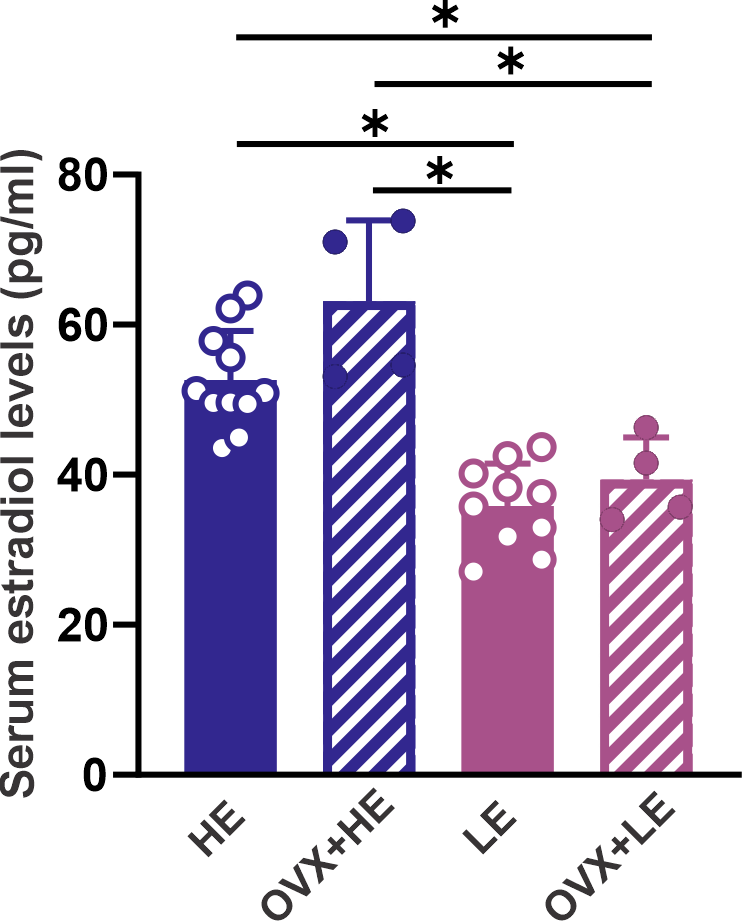
**

**Supplementary Figure 1:** Graphical summary of serum estradiol levels from HE (N=11), OVX+HE (N=4), LE (N=10) and OVX+LE rats (N=4). Note that there is a significant difference in estradiol levels between high and low estrogen groups, but no differences were observed between intact and ovariectomized animals under equivalent hormone conditions (*p<0.05, one-way ANOVA).
